# Immunogenicity of *Anisakis* larvae molting membrane against human eosinophilia sera

**DOI:** 10.12771/emj.2025.00311

**Published:** 2025-04-08

**Authors:** Sooji Hong, Bong-Kwang Jung, Hyun-Jong Yang

**Affiliations:** 1 Department of Parasitology, Ewha Womans University School of Medicine, Seoul, Korea; 2 Ewha Medical Research Center, Ewha Womans University School of Medicine, Seoul, Korea; 3 MediCheck Research Institute, Korea Association of Health Promotion, Seoul, Korea

**Keywords:** *Anisakis*, Molting membrane, Allergic reactions, Eosinophilia

## Abstract

**Purpose:**

This study aimed to investigate whether proteins present in the molting membranes of third-stage (L3) *Anisakis* larvae could serve as potential risk factors for allergic reactions.

**Methods:**

Third-stage larvae (L3) of *Anisakis* spp. were primarily collected from mackerels and cultured in vitro to yield both molting membranes and fourth-stage (L4) larvae. Major soluble proteins in the molting membranes were identified using SDS-PAGE (sodium dodecyl sulfate–polyacrylamide gel electrophoresis). Crude antigens extracted from L3, L4, and the molting membranes were subsequently evaluated by western blotting using sera from *Anisakis*-infected rabbits and patients with eosinophilia.

**Results:**

Antigens derived from the molting membranes reacted with sera from *Anisakis*-infected rabbits as well as with sera from 7 patients with eosinophilia of unknown origin. These findings suggest that unidentified proteins in the molting membranes of *Anisakis* L3 may contribute to early allergic reactions, particularly in patients sensitized by specific molecular components.

**Conclusion:**

Our results indicate that proteins present in the molting membranes of third-stage *Anisakis* spp. larvae may be associated with allergic responses. Further studies are required to confirm the correlation between these membranes and *Anisakis*-induced allergies.

## Introduction

### Background

Anisakiasis is a food-borne parasitic disease caused by the accidental ingestion of raw or undercooked seafood containing third-stage larvae (L3) of *Anisakis* spp. [1,2]. The first reported human infection, presenting with acute abdominal pain due to a marine nematode, was documented in the Netherlands in 1960 [3]. Since that time, anisakiasis has been reported in several countries, with approximately 2,000 cases in Japan, 20–500 cases in various European countries, and 200 cases annually in South Korea—a reflection of the high consumption of raw fish [4]. Furthermore, the global increase in raw fish consumption over the past decade highlights the ongoing need for the management of food-borne infections such as anisakiasis [5]. Human infections with anisakid larvae commonly produce gastrointestinal symptoms including abdominal or epigastric pain, nausea, and vomiting, which are associated with larval migration and penetration of the gastric mucosa [6]. Allergic anisakiasis may trigger mild to severe immunological reactions on the arms and abdomen, and can also result in angioedema or anaphylaxis [7]. Additionally, some patients exhibit generalized hypersensitivity reactions without accompanying digestive symptoms [8].

There are numerous studies examining allergens of *Anisakis simplex*, which have been classified into excretory-secretory, somatic, and cuticular proteins [4,9,10]. However, no studies have yet explored the allergic components of molting membranes isolated from the outer portion of the larval cuticular layer.

### Objectives

We have confirmed that proteins in the molting membranes exhibit antigenicity and cross-reactivity with sera from eosinophilia patients. We hypothesized that not only the main body of *Anisakis* larvae but also their molting membranes may function as key allergic antigens. Therefore, we investigated several antigens from the molting membranes that reacted with sera from *Anisakis*-infected rabbits and patients with eosinophilia linked to unknown antigenic components.

## Methods

### Ethics statement

Mackerels were purchased from local fisheries. Human sera from patients with anisakiasis were collected after obtaining informed consent.

### Antigen preparation

L3 of *Anisakis* spp. were collected from mackerels purchased at a wholesale fisheries market in Korea. The collected L3 were washed with phosphate-buffered saline (PBS), and 2 larvae were transferred into each well of a 24-well plate filled with 0.9% sodium chloride solution to obtain the molting membranes and cultured L3. Three days after incubation in a 5% CO_2_ incubator, the L3 began to molt over the subsequent 7 days, developing into fourth-stage (L4) larvae. Molting membranes were isolated from the larval bodies in the culture medium under a stereo microscope, then washed with PBS and centrifuged at 4°C to extract proteins for immunoblot analysis. The larvae were also incubated at 4°C for 3 days in PBS containing antibiotics to prevent microbial contamination. Antigens extracted from L3, L4, and the molting membranes were prepared using a protein lysis buffer containing Triton X-100 and analyzed by SDS-PAGE (sodium dodecyl sulfate–polyacrylamide gel electrophoresis) on a 5%–15% polyacrylamide gel.

### Collection of antibody

*Anisakis*-infected rabbit sera were stored at –80°C until use in immunoblot assays [11]. At the Department of Allergy and Clinical Immunology of Ewha Womans University Mokdong Hospital, a 3 mL venous blood sample was collected from each patient using a serum separator tube. The sample was stored at 2–8°C until initial allergic testing. The remaining specimen was subsequently transferred to the Department of Parasitology and Ewha Medical Research Center at Ewha Womans University College of Medicine for serum separation by centrifugation at 1,300–2,000 × g for 10–15 minutes. The isolated serum was then aliquoted and stored at –20°C until further analysis.

### Western blot

Western blot analysis of protein extracts from L3, L4, and the molting membranes of *Anisakis* spp. was performed using sera from *Anisakis*-infected rabbits and eosinophilic patients as described above. A peroxidase-conjugated rabbit/human immunoglobulin G (IgG) antibody (MP Biomedicals) served as the secondary antibody, and immune-reactive bands were visualized using 4-chloro-1-naphthol (Sigma).

## Results

**Fig. 1.**
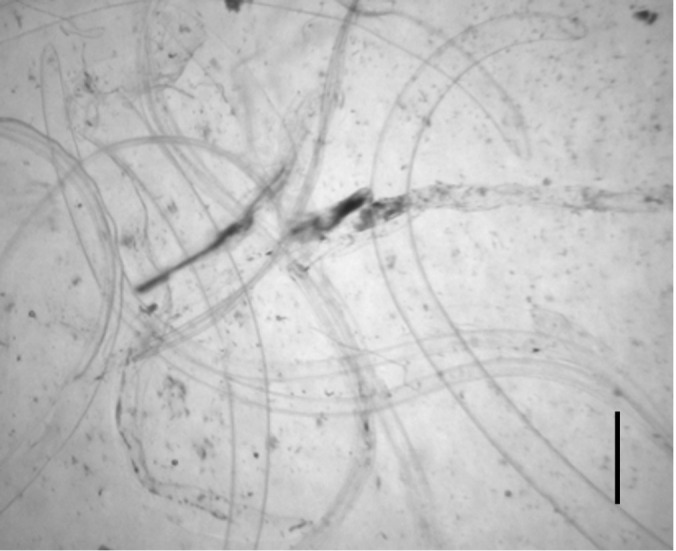
Molting membranes observed after 3 days of incubation of third-stage larvae (L3) of *Anisakis* spp. Note the transparent sheaths seen under a stereo microscope (scale bar=1 mm).

**Fig. 2.**
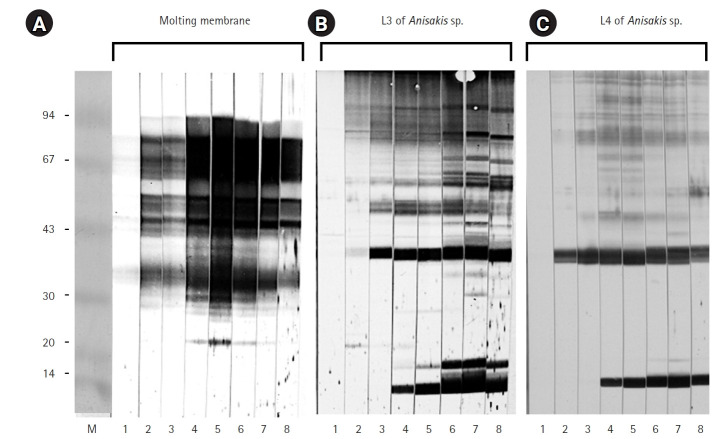
Immunoblot results for the molting membranes (A), third-stage larvae (L3) of *Anisakis* spp. (B), and fourth-stage larvae (L4) of *Anisakis* spp. (C), reacting with sera from *Anisakis*-infected rabbits. Key time points: 1, 3 days; 2, 1 week; 3, 2 weeks; 4, 3 weeks; 5, 4 weeks; 6, 5 weeks; 7, 7 weeks; 8, 9 weeks after infection.

**Fig. 3.**
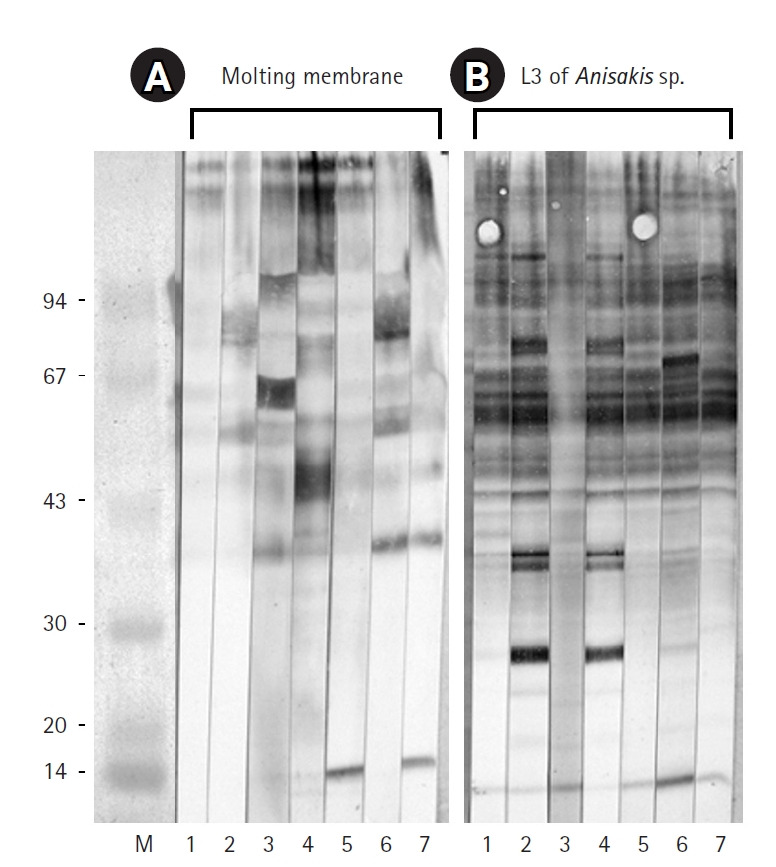
Immunoblot results for the molting membranes (A) and third-stage larvae (L3) of *Anisakis* spp. (B) reacting with sera from 7 human eosinophilia patients. Note the immunoglobulin G reactivity observed against the molting membranes of *Anisakis* L3.

After 3 days of incubation, the transparent sheaths isolated from the bodies of *Anisakis* L3 were identified as molting membranes under a stereomicroscope (Fig. 1). Protein extracts from L3, L4, and the molting membranes reacted with IgG from *Anisakis*-infected rabbit sera, as demonstrated by western blot analysis (Fig. 2). Among the various bands observed in the antigen extracts from *Anisakis* L3 and L4, bands of approximately 40 kDa and 14 kDa emerged at 1 week and 3 weeks post-infection, respectively (Fig. 2). Interestingly, proteins extracted from the molting membranes using Triton X-100 elicited a strong response with rabbit sera from the early stages (1 week) of *Anisakis* infection, producing numerous bands that were not observed in the L3 and L4 extracts (Fig. 2). The antigenicity of L3, L4, and the molting membranes, as detected by reactivity with infected rabbit sera, remained at a high plateau for 9 weeks (Fig. 2). Immunoblot assays performed with IgG from sera of 7 eosinophilia patients revealed an irregular pattern of reactivity with protein extracts from the molting membranes of *Anisakis* (Fig. 3). In contrast, sera from patients 2 and 4, as well as those from patients 1, 5, and 7, exhibited regular reaction patterns against antigens extracted from *Anisakis* L3 (Fig. 3).

## Discussion

### Key results

The molting membranes of *Anisakis* reacted strongly with rabbit IgG during the early stages of infection, displaying numerous unique antigenic bands. In addition, antigens of approximately 40 kDa and 14 kDa from L3 and L4 emerged at 1 and 3 weeks post-infection, respectively. Patient sera exhibited irregular reactivity towards the molting membranes, whereas a regular pattern was observed against *Anisakis* L3 antigens.

### Interpretation/comparison with previous studies

It is well established that *Anisakis* infection can trigger a range of allergic reactions in humans, and research into the allergenic factors of *Anisakis* is ongoing. While the structural and biochemical characteristics of excretory-secretory, somatic, and cuticular allergens in nematodes have been extensively studied, the antigenicity of the molting membranes—isolated from the outer cuticular layer of larvae—remain unexplored. In this study, we demonstrated the strong antigenicity of molting membranes against sera from *Anisakis*-infected rabbits and observed irregular reactivity patterns of these antigens with sera from eosinophilic patients. Our findings suggest that unidentified antigens within the molting membranes may play a role in early allergic reactions occurring within one week of infection. Specific IgG responses to *Anisakis* larvae and their molting membranes were evident from 1 week and maintained at a high plateau for 9 weeks. These results are consistent with previous studies showing that specific IgG production in rats after peritoneal inoculation steadily increased to a high plateau and was sustained for up to 2 months [12]. Moreover, our findings indicate that the diverse antigenic bands present in the molting membranes were absent in L3 and L4 extracts. These distinct immunogenic antigens may have important clinical implications, as they could contribute to allergic reactions. Further research is necessary to identify new major antigens in the molting membranes beyond the already recognized allergens.

Recently, Rahmati et al. [10] in 2020 identified global hotspots for potential allergic anisakiasis, reporting the highest prevalence rates of 18.45%–22.50% in Portugal and Norway. They recommended that allergic anisakiasis be recognized as a significant public health issue in high-risk countries with high consumption of raw fish [10]. Based on this report, we suggest that sensitized individuals may experience hypersensitive reactions upon contact with the outer molting membranes of *Anisakis* larvae, regardless of whether the larvae penetrate the digestive tract.

### Suggestions for further studies

Further studies are required to isolate and identify the major antigenic components of the molting membranes that contribute to allergic reactions. Additionally, investigating the structural properties and immunological mechanisms of these proteins may provide valuable insights for allergy diagnosis and prevention. In clinical practice and public health education, it is crucial to raise awareness about the potential allergenic risks posed by molting membranes—even in the absence of direct larval penetration—especially among high-risk populations that consume raw seafood.

### Conclusion

This study demonstrated that the molting membranes of *Anisakis* spp. possess strong antigenicity and may serve as significant allergens, particularly in patients with eosinophilia. The unidentified proteins within these membranes could play a critical role in the early allergic reactions following *Anisakis* infection. These findings suggest that molting membranes represent additional candidates for eliciting allergic responses and that their identification could contribute to a better understanding, prevention, and management of allergic anisakiasis, ultimately improving clinical care and public health outcomes.
